# Evaluating the implementation of the SWITCH® school wellness intervention and capacity-building process through multiple methods

**DOI:** 10.1186/s12966-020-01070-y

**Published:** 2020-12-11

**Authors:** Gabriella M. McLoughlin, Priscila Candal, Spyridoula Vazou, Joey A. Lee, David A. Dzewaltowski, Richard R. Rosenkranz, Lorraine Lanningham-Foster, Douglas A. Gentile, Laura Liechty, Senlin Chen, Gregory J. Welk

**Affiliations:** 1 Implementation Science Center for Cancer Control and Prevention Research Center, Brown School, Washington University in St. Louis, One Brookings Drive, St. Louis, MO 63130 USA; 2 Department of Surgery (Division of Public Health Sciences), Washington University School of Medicine, Washington University in St. Louis, St. Louis, MO 63110 USA; 3 Department of Kinesiology, Iowa State University, Ames, IA 50011 USA; 4 Department of Health Sciences, University of Colorado, Colorado Springs, Colorado Springs, CO 80918 USA; 5 College of Public Health, University of Nebraska Medical Center, Omaha, NE 68198 USA; 6 Buffett Early Childhood Institute, University of Nebraska, Omaha, NE 68106 USA; 7 Department of Food, Nutrition, Dietetics and Health, Kansas State University, Manhattan, KS 66506 USA; 8 Department of Food Science and Human Nutrition, Iowa State University, Ames, IA 50011 USA; 9 Department of Psychology, Iowa State University, Ames, IA 50011 USA; 10 Extension and Outreach: 4-H Youth Development, Iowa State University, Ames, IA 50011 USA; 11 School of Kinesiology, Louisiana State University, Baton Rouge, LA 70803 USA

**Keywords:** School wellness, Consolidated framework for implementation research (CFIR), Implementation science, Multiple methods, Qualitative, Quantitative methods

## Abstract

**Background:**

School wellness programming is important for promoting healthy lifestyles and academic achievement in youth; however, research is needed on methods that can help schools implement and sustain such programs on their own. The purpose of this study was to investigate factors within and outside the school environment that influenced school capacity for implementation and potential sustainability of wellness programming.

**Methods:**

As part of the School Wellness Integration Targeting Child Health (SWITCH®) intervention, elementary school wellness teams (*N* = 30) were guided through a capacity-building process focused on promoting the adoption of healthy lifestyle behaviors in students. Data on implementation were collected through three standardized surveys and interviews (pre-mid-post) and a post-implementation interview. Indicators of organizational capacity were assessed using the School Wellness Readiness Assessment (SWRA). Paired *t*-tests were run to assess changes in implementation (classroom, physical education, and lunchroom settings), capacity, and stakeholder engagement over time. One-way analysis of variance (ANOVA) tests were run to examine how implementation of best practices (low, moderate, high) explained differences in capacity gains. Qualitative data were analyzed through inductive and deductive analysis, following the Consolidated Framework for Implementation Research (CFIR).

**Results:**

Paired *t*-tests showed non-significant increases in school and setting-specific capacity and implementation of SWITCH best practices over time, in addition to a consistent level of engagement from key stakeholders. ANOVA results revealed non-significant associations between implementation group and gains in school capacity (*F* [2, 24] = 1.63; *p* = .21), class capacity (*F* [2, 24]=0.20 *p* = .82), lunchroom capacity (*F* [2, 24]=0.29; *p* = .78), and physical education (*F* [2, 24]=1.45; *p* = .25). Qualitative data demonstrated that factors within the outer setting (i.e., engaging community partners) facilitated programming. Inner-setting factors (i.e., relationships with administration and staff) influenced implementation. Implementation process themes (e.g., planning, adaptation of resources to meet school capacity/needs, and engaging students as leaders) were cited as key facilitators. Schools discussed factors affecting sustainability, such as school culture and knowledge of school wellness policy.

**Conclusions:**

The results from this implementation study document the importance of allowing schools to adapt programming to meet their local needs, and highlight the strengths of measuring multiple implementation outcomes. Increased support is needed for schools regarding the formation and improvement of wellness policies as a means to enhance sustainability over time.

**Supplementary Information:**

The online version contains supplementary material available at 10.1186/s12966-020-01070-y.

## Background

School-based wellness programming provides an important opportunity to promote children’s physical activity and nutrition behavior [1, 2]. However, factors within and outside the school environment have been found to hinder adoption, implementation, and sustainability of comprehensive interventions [3–6]. To advance research on school wellness programming, it is important to test approaches aimed at building capacity for more effective school wellness programming.

Within the United States, several key legislative actions related to school-based obesity prevention have been enacted in the last two decades. First, the Child Nutrition and WIC re-authorization act of 2004 [7] mandated that by 2006 schools participating in the National School Lunch Program must develop a wellness committee and a written wellness policy incorporating strategies for promoting physical activity and healthy nutrition behaviors, and community and staff involvement. Second, in 2010 the United States Department of Agriculture (USDA) enacted the Healthy Hunger Free Kids Act [8] which stipulates expectations for school nutrition standards pertaining to breakfast and lunches and wellness policy enhancement, increasing the emphasis on schools as health-promoting environments. Finally, in 2016 the USDA introduced the Final Rule mandate [9] which stipulated that school wellness policies must be evaluated every 3 years in regard to the level of policy implementation, and progress to be reported to the school community and stakeholders. These policies mark major advances in school wellness programming potential, yet little is known about how schools are trained and prepared to fulfil such responsibilities in the United States. Further, few initiatives and programs have been developed to provide schools with professional development on building and sustaining programming, highlighting gaps between policy and practice [10, 11].

The School Wellness Integration Targeting Child Health (SWITCH®) intervention adopts a capacity-building process whereby schools are provided with training and professional development on school wellness programming and evaluation practices [12, 13]. Through such a process, school wellness teams (SWT) are provided with autonomy to implement program quality elements (i.e., implementation strategies) and setting-specific best practices (using implementation materials) to the degree that works best for their school and context.

Foundational research on the original Switch intervention [14, 15] demonstrated the potential of multi-ecological-level programming for improving children’s intake of fruits and vegetables, increasing physical activity, and decreasing non-educational screen time. Subsequent work based on an established implementation framework [16, 17] developed and refined the SWITCH implementation process to enable schools to carry out programming more independently [12]. A recent evaluation has documented the specific value of the online platform for building self-monitoring skills in youth [18]; however, there is considerable variability in the use of this platform by the schools as well as their adherence to the setting-specific best practices. Given that the online website, implementation of modules, and integration of resources throughout the school setting represent the key components of SWITCH, the degree to which these are implemented likely contributes to differential outcomes from SWITCH. Further research is needed to understand the contextual factors that influence implementation and subsequent capacity change from the SWITCH intervention. Such information is critical to improving training and implementation materials and providing ongoing support to schools as a means to promote comprehensive school wellness environments.

The dissemination and implementation (D&I) literature offers many approaches and frameworks for understanding implementation with the Consolidated Framework for Implementation Research (CFIR) being one of the most comprehensive and widely used frameworks [19, 20]. The CFIR has predominantly been used in clinical research [21], but recent applications to school-based health interventions have shown value for evaluating implementation of programming [3, 22]. Although CFIR has been used to frame studies testing the implementation of evidence-based programs in schools, a recent systematic review by Cassar et al. [23] highlighted that use has been primarily for interpreting findings, as opposed to guiding study design. The systematic use of the CFIR provides unique advantages to understand stakeholders’ perceptions of capacity and their perceptions related to sustaining programming over time. Sustainability of evidence-based programming has been cited as a key issue in the D&I literature [6] - to advance research on sustainability, the authors specifically advocated for the use of ‘*methodologically stronger primary research, informed by theory’*, (p. 26) [6]. The use of both quantitative and qualitative methods offers advantages for triangulating implementation data, and for identifying areas of dissonance between more objective indicators of implementation [24]. Accordingly, the specific aims of this study were to 1) examine relations between organizational capacity and implementation of recommended SWITCH best practices, and 2) evaluate factors influencing implementation using a multi-method approach.

## Methods

### Study design

We conducted an implementation study using both qualitative and quantitative data to investigate the implementation processes, outcomes, and the factors that influence implementation of SWITCH quality elements and best practices through the lens of the CFIR model [25]. We sought to test implementation of SWITCH as a “real world trial,” one step prior to full dissemination in the Scale-Up Continuum of evidence-based interventions [20]. Consistent with this continuum, the purpose of this implementation evaluation was to “determine the extent to which the intervention is delivered to the target population as planned” (p.3) [20]. The adoption of CFIR and the use of multi-method approach provides advantages for studying the complex relationships influencing school wellness programming. Approval was obtained from the Institutional Review Board (#14–651) at Iowa State University to conduct the project. Consent was obtained from all adult participants through school agreements prior to implementation, and through verbal consent scripts at the beginning of each interview. To ensure quality in reporting of implementation outcomes and qualitative data, we have adhered to the Standards for Reporting Implementation Studies (StaRI) and the Consolidated criteria for reporting qualitative research (COREQ) checklists in this manuscript [25, 26].

### Participants and training procedures

The 2018–2019 iteration of SWITCH involved 30 elementary schools from 22 counties (of 99) in the state of Iowa, located in the Midwestern portion of the United States. Summary data on school characteristics are provided in Table 1. Schools were recruited/enrolled in the summer/fall of 2018 through communication/solicitation and were instructed to form a school wellness team (SWT) of three (or more) school staff members and assign a leader. Training was offered from September to December of 2018 and consisted of a series of preparatory webinars and a required one-day conference at the University campus. The composition of SWT representation for the sample and information on SWT leaders is illustrated in Table 2. The purpose of the webinars was to orient SWTs to SWITCH quality elements and best practices and their importance/rationale as a means to enhance student health behavior (shown in Table 3). This provided SWT with an opportunity to learn the history of SWITCH, the key facets of programming, and all the resources available for implementation. The purpose of the in-person conference was to provide SWT with rich professional development on module implementation, assessing their school wellness environment for strengths and areas for improvement, and strategic planning.

**Table 1:** Demographic information on SWITCH elementary schools (*N* = 30)

Variable	Mean	SD	Range
Enrollment	240.7	112.41	61–506
Percent White	88.0%	19.7	38–100
Percent Low-Income	43.2%	21.54	0–74
Percent Male	52.8%	11.4	40–83

**Table 2:** School Wellness Team (SWT) composition (*N* = 30)

Role	SWT with Representation	SWT Leadership
PE Teacher	24	6
Classroom Teacher	22	7
Nurse	16	8
Food Service	13	1
Principal/Admin	12	4
Counselor	4	1
Instructional coach	2	2
Computer/IT	1	1

**Table 3:** SWITCH® Quality Elements and Best Practices (i.e., Implementation Strategies) with purpose/rationale

Quality Element	Purpose/Rationale
SWT weekly meetings	Meeting as a SWT to plan upcoming activities and implementation; discuss problematic areas
Facilitate online tracking through website	Practicing self-management of health behavior helps students to be mindful of their behaviors and set goals for their own health
Promote student leadership of wellness	Developing a group of student wellness leaders/ambassadors enhances program implementation and may provide students with peer role models for health behavior
Integration of SWITCH across school environment	Using promotional posters to engage students and staff in each setting, use of modules in each setting, and communication with staff outside of the SWT enhances reach and promotion of wellness programs
Communicate wellness efforts to parents	Web-based parent platform linked parents to their student(s) enrolled in SWITCH. Parents could view child’s behavior tracking, enter healthy activities performed at home (“Switches”), view weekly information about creating healthy home environments, and receive SWITCH / healthy updates from the school, helping to transfer lessons learned to the home setting.
**Best Practice** (classroom, lunchroom, physical education)	
Modules	Short lesson plans (i.e., warmups, brain breaks, learning activities) to engage students in physical activity while learning about health & wellness.
Posters	Interactive posters to track Do, View, and Chew activities completed by students across the school settings. In lunchroom and physical education, posters display trivia questions and weekly fun facts related to themes.
Reinforcement of Themes	Utilizing online website for student self-monitoring, use in discussion activities in modules, and making structural changes (i.e., posters, lunchroom modifications). Strategies to help teachers and students learn about health and wellness through a dialectic approach.

Note: *SWT* School wellness team

Moreover, in the 2018–2019 iteration, a key focus for implementation was to establish reciprocal and collaborative relationships with a national youth development organization: 4-H [27] through the Iowa 4-H Extension and Outreach program [28]. Extension staff, that facilitated county-level 4-H programming, were encouraged to attend the conference and meet with SWT from schools in their county to learn about SWITCH and plan programming collectively. The 4-H Extension staff facilitated goal setting through provision of ideas and support, and worked with SWT to develop an action plan for the 12-week implementation phase and beyond. A separate evaluation of 4-H Extension engagement and program satisfaction was conducted; it highlighted the ways in which Extension staff felt that SWITCH provided them with an opportunity to reach more schools and youth in their counties, indicating a mutually beneficial relationship for both extension/4-H staff and schools SWT [29].

### School capacity measurement

The SWITCH evaluation has incorporated indicators to capture elements of organizational capacity or readiness as well as environmental characteristics [30]. A specific tool called the School Wellness Readiness Assessment (SWRA) [30] was developed based on previous conceptions of organizational readiness [31]. It consists of questions to assess readiness in the overall school setting, as well as classroom, lunchroom, and physical education settings. SWT were asked to respond on a 5-point Likert scale ranging from “Strongly disagree” to “Strongly agree”. The measure was developed through multiple rounds of review by experts in school wellness and had documented utility in predicting engagement with the launch of SWITCH in the previous iteration of SWITCH (unpublished observations). Each construct has high internal consistency; classroom α = .85; PE: α = .85; lunchroom: α = .94; and school organization: α = .93 [30]. The SWRA was administered prior to implementation (week 0) and again at the end of implementation (week 12+). The tool can be found in Additional file 1: Appendix A.

### Implementation measures

#### Checkpoint surveys

Checkpoint surveys were administered halfway (week 6) and toward the end (week 12) of implementation to assess the degree to which SWT were able to implement the SWITCH program. Wellness teams were asked to report the degree to which they adhered to the five quality elements established to guide schools (see Table 3). They also reported on setting-specific implementation by reporting on adherence to similar best practice guidelines recommended for the classroom, lunchroom, and physical education settings. Both sets of items were coded into three levels (Not Yet/Partially/Fully; scored as 1, 2, or 3, respectively) but were combined as a global indicator for the present study to capture information on Implementation Process (Planning, Executing) factors in the CFIR. Previous research supported the importance of an individual quality element (use of the web-based tracker) as a predictor of changes in youth outcomes [18] so this provides some documentation for the overall utility of these items. Additional questions in the checkpoint survey asked about the perceived engagement and awareness of school stakeholders (i.e., classroom teachers, administration, food service staff – 1 = low; 2 = moderate; 3 = high) to gain a sense of how the SWT are networked within their school community and the degree of collaboration within and outside the building.

#### Checkpoint calls

Checkpoint calls were utilized before and throughout the implementation phase to connect with schools and facilitate goal setting/monitoring. These calls were introduced in the 2017–18 cycle of SWITCH as an implementation strategy designed to facilitate adoption and implementation of programming by helping SWT hold themselves and their colleagues accountable during the 12-week phase. They were maintained as a key element of the training and implementation process in the 2018–19 cycle as the research team was able to engage 4-H Extension staff to conduct these capacity-building calls [29]. Checkpoint calls were scheduled before implementation (week 0), half way (week 5/6), and toward the end (11/12) of the active implementation phase. Each call was grounded in motivational interviewing (MI) principles [32, 33], whereby researchers (*n* = 2; masters/PhD-level education) and trained 4-H Extension (all with bachelor’s or master’s degree) staff posed questions to SWT with the goal of guiding them to a strategy/solution that works best for their team capacity and school environment. Field notes were recorded during the first interview as a means to document goals set by the SWT and to help hold schools accountable for their progress [34]. In doing so, support to schools was individualized and specific to their needs as an implementation site. Schools were asked to allot ~ 30 min before/during/after the school day for these conversations. All schools had met the researchers and Extension staff at the training conference in the fall prior to spring implementation, and were aware of the supporting role of Extension staff prior to involvement. Questions asked in these calls pertained to the Innovation Characteristics (Adaptability, Complexity, Cost), Implementation Process (Planning, Engaging, Executing), Inner Setting (all constructs), and Outer Setting (Cosmopolitanism, Peer Pressure) factors which attributed to SWT’s ability to implement SWITCH and develop comprehensive programming [19]. All calls were audio recorded and transcribed. All interview guides can be found in Additional file 2: Appendix B.

### Semi-formal interviews

**Fig. 1:**
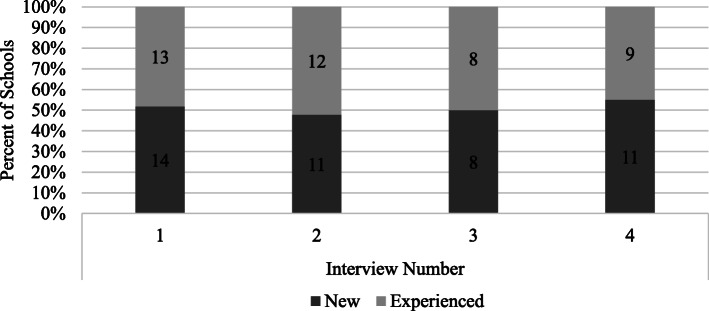
Number of schools that were interviewed at each time point, by SWITCH experience. Note: 1 = pre; 2 = mid-point; 3 = week 11/12; 4 = post

After implementation concluded (post-week 12), a semi-formal interview was conducted with SWT by an independent contractor, highly-skilled in qualitative methods, to gather reflective input and feedback on the program and the ways in which schools were able to perceive their ability to sustain wellness programming after formal implementation ended. The interview guide was designed to address how training and support from the SWITCH research team/Extension, as well as factors within/outside the school environment, affected SWT ability to implement the program. An example question was: “How would you describe the level of support you received from your county youth Extension officer(s)? To what degree did they facilitate SWITCH implementation?”. Interview data provided valuable insights that could not be gleaned from checkpoint calls, as SWT reflected on all 12 weeks and provided objective feedback on the program [34]. Interviews lasted between 10 and 27 min, conducted through phone call or online video conferencing software (i.e., Zoom), were audio recorded, and transcribed verbatim. All SWT were sent an email from the SWITCH research team and Extension officers to schedule checkpoint calls; final interviews were scheduled with an external research contractor through email contact. Figure 1 illustrates the number of interviews/checkpoint calls conducted at each time point, showing an even distribution between new and returning schools. School data were collected from only those SWT that responded to invitations for checkpoint calls and interviews. Not all SWT participated in interviews.

### Data analysis

All quantitative data were entered into Statistical Analysis Software (SAS; Version 9.6, Raleigh NC), processed, and tested for normality of distribution. Checkpoint survey and SWRA data were merged with school demographic data and sorted by time, providing two time points (pre- and post-SWITCH) for each school. Given the dearth of school-based D&I research to date, guidelines for how to analyze implementation scores from multiple indicators was drawn from public health and clinical fields [35, 36]. The five quality elements of SWITCH: SWT weekly meetings; using the tracking software in classrooms; integration of curriculum in each school setting; engaging youth as leaders; and communicating with parents have all shown to be important implementation steps to facilitate school-wide change and/or subsequent youth behavioral outcomes [12–14, 18]. Since these practices hold ecological validity for outcomes of the SWITCH intervention, they were combined into a global implementation score. To establish a composite measure of implementation, an average quality element score (5 elements; scored 1–3) was added to average scores for each of the setting best practices (lunch, physical education, classroom; scored 1–3), yielding a score between 2 and 6. Subsequent tertile splits were computed to separate high versus moderate and low implementing schools; such method has strong support and utility in modeling research especially when a linear relationship cannot be assumed among variables [37]. Scores were calculated for overall SWRA scores, and changes in capacity in each school setting (post-pre) were computed to determine capacity change over time.

Pearson bivariate correlations were conducted to examine relations between changes in implementation over time and changes in school-level wellness capacity. In addition, paired *t*-tests were conducted to examine changes in setting-specific capacity over time. Finally, a series of one-way ANOVAs were run to understand the differences between implementation (low, moderate, high) and subsequent gains in capacity (setting specific and overall). Statistical significance was assumed at α < 0.05.

Qualitative data collection yielded 90 transcripts across the four time points, requiring a rigorous and systematic procedure; thus, analysis followed constant comparison techniques with two coders [38]. Based on this approach, data were analyzed first through open coding, followed by axial coding while maintaining an inductive approach, before deductive analysis was conducted through the lens of CFIR domains. This provided a clearer understanding of how factors related to the intervention and those within and outside the school environment affected implementation and SWT capacity to achieve systems change in their school community. The purpose of axial coding was to test the un-coded data against the initial codebook for agreement and to make refinements to themes [39, 40]. A separate subsample of transcripts from each time point were reviewed and coded according to existing themes. Where new ideas/themes emerged, the coders developed new nodes in NVivo to accommodate emerging concepts. Multiple first-order themes were generated with several second order themes encapsulated within these concepts. The final product of this phase was a more comprehensive codebook with many main (> 10) themes and subthemes that represent qualitative data.

Deductive analysis comprised a systematic approach to conceptualize how the data aligned with CFIR. Use of the comparative method and negative case analysis were highly integrated, as it was important to look for cases that do not align with the overall implementation framework structure [39, 40]. When initial themes were developed, the lead author and another co-author reviewed the themes and began to merge the inductive codebook with the NVivo CFIR codebook template [41]. This process allowed the researchers to carefully consider each domain and construct, and to determine appropriate alignment to the model, where relevant (Additional file 3: Appendix C). Furthermore, researchers sought to code data related to the sustainability of SWITCH and the degree to which SWT believed they could continue programming as part of school culture.

Guided by experts in qualitative analysis [42, 43], a series of strategies were employed to ensure the trustworthiness of the data and analyses. First, a comprehensive audit trail was kept throughout the analytical process between the two primary coders to document key issues, trends, and whether any data saturation occurred, facilitating transparency in collaborative research [44]. Second, peer debriefing took place whereby two other researchers reviewed codes and provided feedback on the interpretation of data, identifying gaps or areas of potential oversight [43]. Finally, thorough negative case analyses were integrated to ensure that themes accurately represented the data and identified areas of divergence from themes [42, 43].

## Results

### SWITCH implementation

**Fig. 2:**
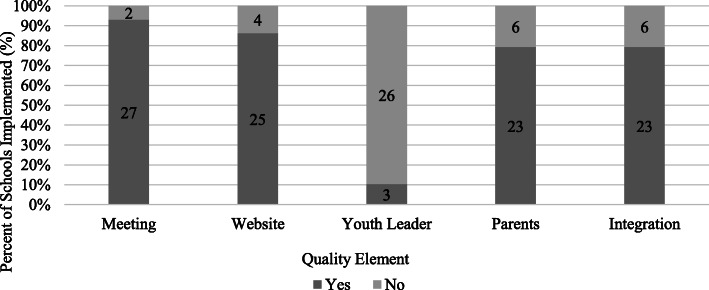
Implementation of quality elements at half-way point (Week 6)*; *N* = 29. Note: *Quality element implementation only measured at ~half-way point, Meeting = SWT weekly meetings, Website = facilitate online tracking through website, Youth leader = Promote student leadership of wellness, Integration = Integration of SWITCH across school environment, Parents = Communicate wellness efforts to parents. All numbers represent the number of schools who reported implementation of this best practice

**Fig. 3:**
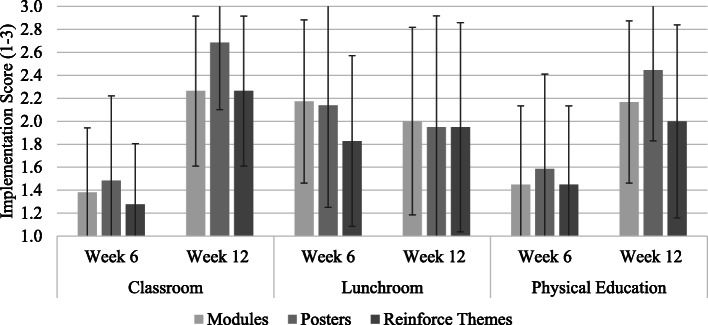
Implementation of setting-specific best practices at mid-point (week 6) and end-point (week 12) of SWITCH

Overall, SWT reported implementing most of the program quality elements (see Fig. 2). Almost all schools reported regular SWT meetings (*n* = 27, 93.1%), followed by getting students online and adopting self-monitoring skills through the website (*n* = 25, 86.2%). Comprehensive integration of using SWITCH resources across the school setting (*n* = 23, 79.3%) and working to involve parents (*n* = 23, 79.3%) were implemented fairly well. Only 3 schools (10.3%) reported involving youth in a leadership capacity to deliver wellness programming (see Fig. 2). For setting-specific best practices, at week 6, implementation in the classroom was lowest compared to the lunchroom and physical education. In week 12, the opposite occurred, whereby classroom implementation was the highest, followed by physical education and lunchroom. The lunchroom implementation scores all decreased aside from reinforcement of themes (see Fig. 3).

**Fig. 4:**
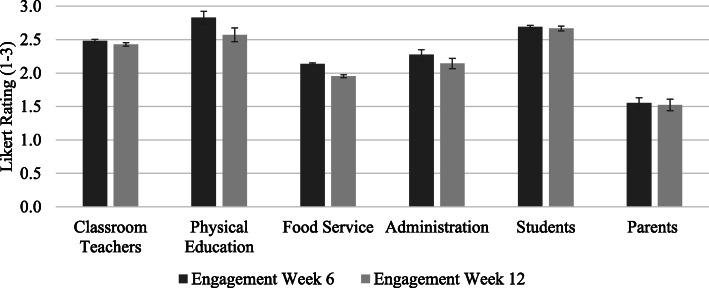
Engagement of school stakeholders at mid-point (week 6) and end-point (week 12) of SWITCH. Note: Rating of 1 = not at all engaged; 2 = somewhat engaged; 3 = very engaged

**Fig. 5:**
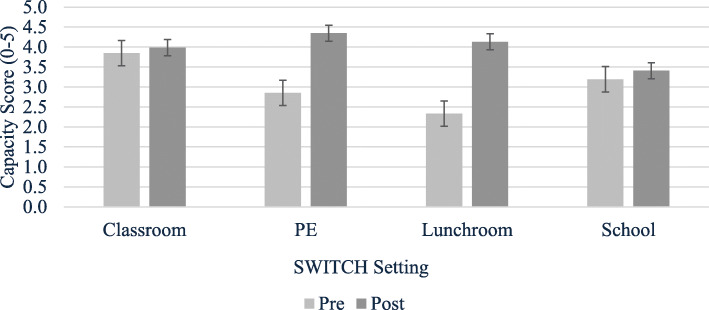
Changes in setting-specific capacity over time

The SWT rated the relative engagement of key stakeholders in SWITCH at several timepoints (Fig. 4). The reported engagement at week 6 and 12 was highest among physical education (2.9 ± 0.4 and 2.8 ± 0.5, respectively) and students (2.8 ± 0.4 and 2.7 ± 0.5, respectively) and lowest for parent engagement (2.0 ± 0.6 and 1.5 ± 0.5, respectively). All scores decreased slightly, except for student engagement, which remained the same. The SWT reported increases in capacity over time, as captured by the SWRA tool, in each setting (see Fig. 5). The biggest increase in capacity was in the lunchroom, from 2.3 ± 0.5 to 4.1 ± 0.5, equaling a 78% change in score, followed by physical education (from 2.9 ± 0.4 to 4.3 ± 0.5; 48% increase). School capacity increased 6% (from 3.2 ± 0.9 to 3.4 ± 0.5) and classroom capacity increased 2.5% (from 3.9 ± 0.6 to 4.0 ± 0.5), with classroom having the highest capacity at baseline.

**Fig. 6:**
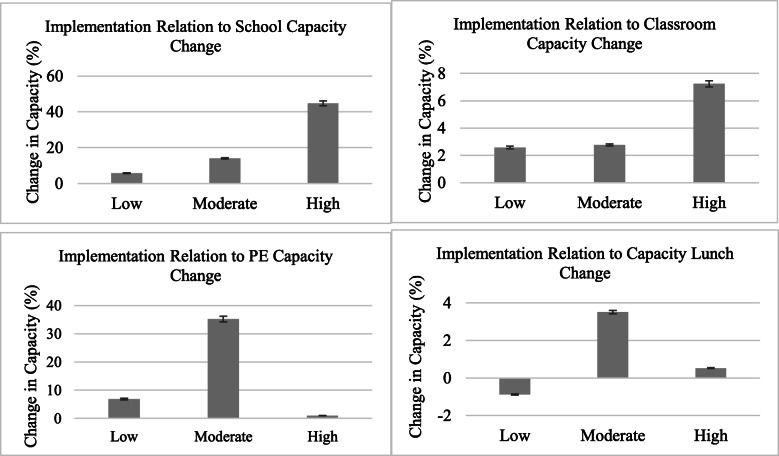
Findings from one-way ANOVA analyses on the relationship between implementation level and capacity change. Note: Effects and significance for school (*F* [2, 24]= 1.63, *p* = .22); classroom (*F* [2, 24]= 0.20, *p* = .82); physical education (*F* [2, 24]= 1.45, *p* = .26); lunchroom (*F* [2, 24]=0.29, *p* = .75) capacity change as a function of different implementation groups. Bars represent adjusted LS Means with standard error (SE) bars

Pearson bivariate correlations revealed non-significant associations between implementation composite scores and percent changes in capacity for the school (*r =* .16), classroom (*r =* .35), physical education (*r =* .02), and lunchroom indicators (*r =* .06). Paired *t*-tests also showed non-significant results for setting-specific capacity changes over time (all *p* > .05). ANOVA results also showed non-significant findings. As Fig. 6 shows, small but non-significant differences were found regarding levels of implementation (low, moderate, high) for percent in capacity change for school (*F* [2, 24]= 1.63, *p =* .22) and classroom (*F* [2, 24]= 0.20, *p =* .82) settings, with schools in the high implementation group reporting greater capacity change than the middle and low groups. For physical education (*F* [2, 24]= 1.45, *p =* .26) and lunchroom (*F* [2, 24]=0.29, *p =* .75), tests show that the moderate implementation reported higher but non-significant capacity gains compared to both the low (and high) implementation groups.

### Qualitative themes

Table 4 displays all themes, grouped by CFIR construct and domain; selected quotations from the responses are added to illustrate each theme, providing context and negative cases where relevant.

**Table 4:** Qualitative data themes from School Wellness Teams (SWT) linked to CFIR thematic structure

Construct	Theme Description	Salient Extracts from Calls and Interviews
**I. Intervention Characteristics**		
**Adaptability**	Adaptation of modules for school needs	“We started talking about the food groups and energy. We talked about food chains and giving the food energy for the consumer. I tie it all in that way which really helped, and we’re um, next week we’re going to be hitting the respiratory system so I’ll tie in some of the switch lessons.” “With the food service, I think I think we’re getting more tasting foods, new foods. But you know, she’s limited on her budget to what she can do. So, we’ve had people bring things in.”
**Complexity**	Perceived complexity of the intervention	“Our staff has already been trained on the benefits of movement during class. It’s more a refresher of other ways to incorporate movement while learning. It doesn’t necessarily just have to be a straight break. So, we’re going to provide a few doses of training throughout the rest of the year.”
**II. Outer Setting**		
**Cosmopolitanism**	Extension support	
	***Subtheme****: Part of the team*	“[Extension] has brought [SWITCH] to professional development, she has put an article in the newspaper, so she is always talking about it, she’s bringing things up um, and so I really feel like our staff in on board.”
	*Provision of ideas and resources*	“[Extension staff], I just want to tell you that your brain breaks that you guys sent us…. Our students did ask for these about every day.”
	*Accountability of goal setting*	“I think we were trying to go more with the flow and trying to rely on our expertise so I think the things we got from [Extension] were probably more helpful because she knew what our goals were. It was focusing on certain things that we were working on here.”
	*Negative Case: Lack of extension support (5 SWT reported)*	“I didn’t know how available [extension staff] with the [university] extension was. You know, it said she was available but never told us to what extent. And because of that, I didn’t know how much time I could use her.”
	Collaborating with community/organizations	“We’ve had a couple of try-day Fridays where the kids are introduced to three new [food] items…We were able to get the donations for that which was nice.” “We have the farmers market come to us. In the parking lot and each kid gets so much money and they actually go out and purchase things from the farmer.”
**Peer Pressure**	Positive peer pressure from other schools	“I know if like the other schools [at the conference], this is their second year that the success rate is significantly higher, because the first year is super trial and error, where you’re just like stumbling through it, and forgetting you’re doing it. It made us say, ‘Oh yeah, we’re doing this? We’ve got to do something!’”
**III. Inner Setting**		
**Networks & Communications**	Greater input and implementation from those outside the SWT	“I think with SWITCH it kind of makes us more aware of our wellness policy and we’ve started working more closely with [PE Teacher] because he’s here at like a couple hours in the morning so I don’t typically see him ever and so, you know, if you don’t see somebody you don’t think about it and you never talk to them. And, so, like he’s been more a part of SWITCH this year for us and I don’t know why we didn’t think about this last year.” “I feel like across the board teachers are realizing more and more that they need to be getting up and moving these kids around.”
	Lack of staff buy-in	“We purposefully brought our food services gal to the conference, because we knew that that was a big challenge. She was kind of willing to go, but now since has not been too willing to change much.”
**Culture**	SWITCH experience facilitated implementation and buy-in	“This will be our third year that we do it at the Elementary and slowly but surely I think I’m getting more teachers on board to recognize the value of wellness and increased opportunities and stuff.” “It’s nice to have a year underneath our belt, and that, just the catchy phrase of DO, VIEW, AND CHEW, I mean, It’s easy for the kids to, uh, latch onto. I know our Try-Day Friday was probably the biggest hit.”
	*Lack of prior wellness involvement posed challenges*	“One thing that we had talked about in our meeting the other day was um, the school that is still in our district but they had already done it are already talking about doing a fitness night with the parents…We’re not sure we’re ready to tackle that in our first year, but we have talked about maybe doing, having the 4 classrooms, and doing like a Do, View, and Chew [lesson] in each classroom, and the kids rotate.”
	Overwhelming staff	“I think [5th grade teacher] probably felt overwhelmed, because she took care of all fifth graders. And she was good at her lessons. Um, an- and I think that there needs to be more... instead of just a couple people like her. Really the teachers all need to buy into it.” “I think this year ... It seems like every year I’ve been here, we’ve just been busier and busier. I think every year they seem to throw more and more stuff at the teachers that they have to get done in class.”
Compatibility	Tension over changing the “status quo”	“[Food service director] has been here a long time, she’s in her ways. She’s not against taste testing, as long as we do it outside of her area, and have somebody else do it, and it doesn’t interrupt her ... they’re just very set in their ways, so that’s our biggest challenge.” “Teachers have a lot of concerns about [activity breaks]: “but [activity breaks are] just going to make them rowdy” and “that’s just gonna get them hyped up and they won’t calm down and sit down” and that might initially be true when we start them and so maybe that would be a good goal for us.”
Relative Priority	Active promotion and priority of SWITCH	“Well for our wellness environment we’ve added, um, another block of recess time. Every day from, 2:30 to 2:40 we just added that and it’s, not saying they’re naughty but like their energy levels seems to be more focused just by, it’s like taking that ten-minute break, the afternoon goes by so much better.”
**Leadership Engagement**	Administrator support	“I think just the kickoff aspect of it, and have a note going home, uh, our principal sends out a note each week to the families, what’s going on, and make sure everybody knows that, uh, next week’s the kickoff, and if they hear their child talking about it, they can expand on it.”
	*Negative case: Poor administrator support (6 SWT reported)*	“I think it’s getting some of those messages at the forefront. I know that for me it’s a struggle to get my administration on board and so I feel like I’m ... I just feel like it’s a struggle all the time to really have to fight to get in the classrooms and get time.” “We just don’t have the time right now with no school, I mean you know…And our administrators don’t want to sound certain lessons.”
**Available Resources**	Lack of resources	
	*Time*	“The thing that I find the most challenging is, we don’t have time to teach what we’re supposed to teach. And this is now on top of that. That’s the biggest challenge I have.”
	*Facilities/Personnel*	“And I’ve also been talking with our PE teacher and the nurse, and I brought it to the school leadership team’s attention that we want to try and do something like the walking program in the mornings, but we’re very limited in our new space. We have no playground, per se.” “It’s a part time school, [lunchroom staff] are employed by the school, but they’re part time. So, they’re on from this time to this time, so they don’t want to be anything extra because they don’t get paid for that. So, having them on, you know on a committee, on this ... you know it doesn’t work very well.”
Access to Knowledge & Information	Online platform usability	“Fourth graders did not do any tracking [online]. The very first time, um, [teacher] said he did it, it took him 45 min of class time. And he says he just couldn’t do it again.” “To record their information in and... it’s hard for them to get on the computers every single day to log in. So, we did a paper form last year and then once a week they would log in.” “I was a little bit when it first started…we share chrome books and sometimes their Internet wasn’t up and powerful as it needed to be and now that we had to share it.”
**IV. Characteristics of Individuals**		
**Individual Identification with Organization**	Core team - school communication and perceived support	“Yesterday during PE, [food service] brought in the lunch trays. As I’m having PE! They just wheel them in and stick them and leave them in the gym. I’m like, “Class is in session.” So yeah, some logistics...”
**Other Personal Attributes**	School wellness team (SWT) motivation	“My kids were with us, so, um, I sent [newsletter] out to all the teachers today and said “I would love to bring my speaker in to every classroom and show your kids this.” And so, I’ve already got [an activity break]. I’ve got a schedule already started working on. So, I’m going to take that ... and I mean, it’s a fun song, and if you play it loud, I think they’ll love it, so. These little tiny things that take two seconds ... I mean, it takes three minutes total. It’ll be fun to integrate not only our fourth and fifth graders, but actually our [whole] school.” “We are- we’re trying very, very hard, I just want you to know. We are trying to do this on top of all of our regular responsibilities. So, school counselor, school nurse, and PE teacher for the district. Um... so we’re trying to make it as easy as we can on ourselves. So, if what we’re doing is not meeting the requirements, tell us. Otherwise, we want to do the very bare minimum.”
**V. Process**		
**Planning**	SWT weekly meetings	“I think the other thing that’s a challenge that for us is that two of us on our, on our team are part-time, so [teacher] and I are part-time… and then [assistant] does before-school activities, and then after school, he’s busy too… a challenge we’ve had, is trying to figure out when’s the best time for us to meet and making sure that we don’t forget those. We don’t think it’s feasible for us to be able to meet on a weekly basis, but we’re going to try to meet every other week and see how that goes.”
	Striking a balance- avoiding burdening staff	“I think [PE teacher] has his information, he’s ready to go, he’s used to doing all this kind of stuff. I think our biggest challenge is in the classroom. Trying to get one person to try to help the other two teachers put it together and, as being a school nurse, I just don’t feel comfortable with it. I’m an educator, too, but I don’t know where I can help because I feel that a lot of SWITCH is really being put on the classroom teacher.” “I think the lessons are pretty well laid out um, Jen took the time to go through and, each lesson has a folder that the teachers can come in, grab, and are welcome to use whenever they can get that in.”
	Importance of training conference	“It helped having our students [at the conference]. I was able to understand it more because I went this year because I didn’t go last year. It really helps the new people. It helps to listen to people’s ideas that they shared what worked at their schools.”
**Engaging**		
Opinion Leaders	Changing students’ perceptions of health behavior	“I can speak for my class with what they’ve done. I’ve got a lot more that are open minded when it comes to trying things, whether it’s, you know activities, or whether it’s the foods. When we talk about, when we do our nutrition lessons and we talk about things I don’t hear as many, grunts and groans as the things, as lessons went on about, you know, food choices.” “I watched them! Like never before have they eaten red peppers at lunch, but they ate ‘em yesterday! Isn’t it crazy when you introduce it like, at a certain time, you know, then it like, it does. I brought, um, grapefruits to [another school] yesterday, and the kids were all like, “No, we’re not eating them.” Then they were like, “We want more!”
	Making parents aware of SWITCH	“I think things are happening, because I saw [a parent] at teachers’ night, she was like, ‘Oh, my daughter just asked me to buy different fruits and vegetables at the store.’” “I actually had a mom that emailed me today saying how her son has talked about the smoothies so she wanted to know if I could give up the recipe.” “It’s very hard. You know, we have the language barrier and a lot of our parents work… we have a large number of parents that are not engaged in what’s going on here at school.”
Formally Appointed Internal Implementation Leaders	Shared versus sole leadership	“Just so it’s not just the two of us always making the decisions for everybody, because that’s when where we felt like we would do something, and then it would just be like, well. It didn’t seem to be well received, and I think if we get everybody involved it would be a little bit more well received.”
Champions	Within SWT coordination	“I think what hurts us is me being here just half a day. Um because then it’s hard to follow up with Angie and get together with her because it’s just, you know, if I were here all day, then there’d be before school and after school that her and I could cross paths um so I think that’s been kind of a barrier. But overall I think it’s been successful.” “I’m trying to keep it going, but it’s, it feels like some days it’s dying a slow death…Well, it’s because it’s so hard, um, you know. I keep thinking, “Gosh, if we had a nurse here, that was here all the time, that could kind of help spearhead [programming]. I mean [nurse] been great about, you know, willing to, to help out and everything, but busy with all her hats that she wears and it’s hard when you don’t have that dedicated person that can really just take it and run with it.”
	Student leadership in programming	“We’ve gotten our youth ambassadors more involved since the last time we talked. They’ve made another video. They’ve really gotten on board with being part of our SWITCH booth at parent teacher conferences. They’re planning to do some activities, and um they made the flyer to give to the kids and that was their idea.” “And I think we’ll see that kids leading the teachers then. Because if you give a wellness coach of the week title, they will not neglect their duties. They will remember that.”
External Change Agents	Collaborations with community	We did a meeting with [dietician] from [local grocery store]. It went really well. She’s going to come visit the school and she gave us some ideas for our tastings and we were gonna do a demonstration at parent-teacher conferences…. She’ll just have some food out there for them to try. We did that last year and it went pretty well.” “Our AmeriCorps person will be in the classroom next week doing nutrition and then our school nurse, she and I are going to do a little blurb on dentistry, oral health, things like that. And that’s because we had some data come up in our district after we had hygienists in the building.”
	County extension leaders running programs	“She made us stickers to go in the assignment books that we had for the students. They could document how many hours of screen time they had, how many fruits and vegetables they had and how many hours of activity. We also documented how many steps, we have pedometers and so we also documented how many steps we had and we used those stickers for that.” “[Extension staff] actually came and talked to our staff about what we had been doing and how we were working together. She came and participated in parent-teacher conferences. Her and the guidance counselor set up a station together downstairs for the parents as they were coming in.”
**Executing**	Module implementation	“We don’t have much PE time. Our kids have one PE class a week usually of 40 min… So, for [PE teacher] to do these activities plus regular PE is, I would think, a challenge.”
	*Posters and student incentives*	“Having the posters and stuff up in the gym, or in the, you know, lunch room is very easy to do. That’s easy to maintain, the kids see it all the time”
	*Adaptive implementation strategies*	“I was pretty enthused about it because I thought it kind of brought all three things together with the do, chew, and view. So many times, it seems like people want to concentrate on just one of those, but it’s a combination of all those that really makes a difference. As a PE teacher with the lessons, those weren’t any problem to implement. I modified most of them to fit our situation.”
	New wellness programs and structural changes	“In the mornings, in the winter time, we’re doing yoga, before school starts.” “[Before SWITCH] The milk was just worded in the same, you know, usually the chocolate and the white are both up sort of in front not really separate. White in the front, chocolate in the back. Um, so that week it was probably about 65% were choosing the white over the chocolate. So then [lunchroom staff] showed the amount of sugar that’s in the white versus the chocolate and what’s the better choice and what’s the healthier choice…. and after the lesson, it went up to 85%.”
	Environmental barriers/weather issues	I think we might want to, at least push it off just because we’ve had so few days of school that I think, I mean teachers are already freaking out about not having their kids in class.” “As far as any classroom time, I have not been in the classroom because every time I’m supposed to be in the classroom, it’s a snow day.”
	Staff wellness initiatives	“I had a chart by my office that if they did sixty minutes of exercise during the week, at least minimum, then they could put their name up on the chart, and it was kind of a challenge. The kids saw it when they went out for recess to see if their teacher was doing it. And we had one faculty staff member that did it all twelve weeks, so we gave her a salad cutting bowl.”
**Reflecting & Evaluating**	High potential for sustainability	“It really helps and then we incorporate more brain breaks in the afternoon, I think that’s even better. Because they get up and move, you know, every few minutes and for the sustainability, this isn’t going to be too difficult to sustain at all.”
	*Practice to policy- Wellness policy improvement*	“Last year we had our nutrition audit and I’m the Food Service Director as well as the School Nurse, and um, we added the Switch program to our Wellness Policy, and that fit in perfectly and they were very impressed that we were doing that. So, that was really nice to have that on board and in place when they came to do our audit.”
	*Lack of policy change, awareness*	“Part of it that I struggle with and so does [colleague] is the school policy because administration and they say it was reviewed and approved at board and [colleague] didn’t even know it was reviewed. And we’re like well, we don’t do this. Does it follow? So, I think to them it’s just a document on paper but I also know their focus since last year was getting the referendum to pass, so they weren’t listening to anything that we were suggesting. So now that it’s done and in progress, perhaps we can approach them about it.”

#### Innovation characteristics

Findings from interview and checkpoint call data highlight the ways in which SWT adapted SWITCH best practices and resources to fit the needs of their school system. One of the examples commonly cited was the adaptation of module/curricular content intended for one setting into another setting, as illustrated in Table 4, where the classroom module was adopted into science curricula to synergize with concepts taught in this content area. Further, schools discussed how they adapted activities, such as conducting taste tests, on a limited budget, demonstrating the ability of the intervention model to be adapted to school needs and capacity.

Many SWT discussed how taking part in SWITCH forced them to consider how to implement all its parts, which required extensive planning and converging at the in-person training conference and the weeks thereafter. In relation to the theme of complexity, several SWT saw the need to revisit how classroom instruction was conducted and took it upon themselves to provide professional development and resources to their peers as a means to enhance the implementation.

#### Outer setting

In 2019 the partnership with state 4-H Extension grew and the collaborating staff were encouraged to support school wellness initiatives by meeting with schools and helping with implementation, relating to the construct of cosmopolitanism. This support ranged in its intensity; some SWT felt that Extension support was vital to their success, stating that their local 4-H staff were a “part of the team” and took responsibility for various tasks. Other SWT talked about how Extension staff kept them accountable for the goals they had set in the first checkpoint call and were responsive to requests for help with various programming aspects.

Schools also collaborated with local agencies, predominantly for help with supply of food and equipment for programming and for hosting certain school-wide events for students and families. Finally, in relation to peer pressure, some schools reported hearing about the ways in which other returning schools had been successful in the 2017–18 SWITCH cycle at the in-person conference, and about what other schools in their district were doing, using this as motivation for planning their wellness activities/programs in the 2019 cycle.

#### Inner setting

Concerning networks and communications, most SWT discussed relationships with other teachers outside the SWT as a factor in their perceived ability to execute SWITCH best practices; most reported a positive reaction to programing and that their teachers were getting on board, whereas others mentioned a lack of staff buy-in which hindered their efforts. For organizational culture, there was a sense of staff members feeling overwhelmed with other responsibilities, which may have attributed to the lack of buy-in from the whole school.

Most SWT reported good support from administration (leadership engagement), and in relation to relative priority, some SWT reported that they were able to schedule additional opportunities and make room for wellness in their schedule, such as adding another block of recess. Unfortunately, some SWT lamented the lack of time provided to plan as a team and lack of facilities to conduct programming (i.e., available resources). Finally, although SWITCH was viewed positively by most staff outside the SWT, some SWT discussed the challenges faced when trying to change unhealthy “norms” in school culture, such as using recess as punishment, food as a reward, or more veteran staff not wishing to innovate in certain areas of the school setting which indicates a lack of compatibility. These factors acted as barriers to implementation of programing and relate to other findings (e.g., lack of buy-in, limited time/resources, etc.).

#### Individual characteristics

Throughout the formal programming phase, it became clear that other personal attributes such as SWT motivation was varied, as some teams were more motivated/excited than others. Table 4 reflects some of the key extracts from SWT, highlighting the divergent perspectives and situations. Most of the low motivation was linked to SWT feeling overwhelmed, and was reported predominantly in inexperienced schools, suggesting that lack of motivation/excitement may be attributed to inner setting factors (see inner setting- culture for additional information). Further, with regard to identification with the organization, SWT felt a strong sense of collaboration and engagement from others in their school, but some discussed sociopolitical tension in their school setting, which impeded their perceived ability to implement programming across the school setting.

#### Implementation process

For the planning construct, the in-person training conference was cited as a key socialization factor and motivator for school teams, particularly new schools, as they were able to learn from other SWT about their implementation practices and launch goal setting/planning for the semester ahead of time. Throughout the implementation phase, many schools discussed ways they were able to meet and accomplish planning responsibilities while balancing different schedules, such as biweekly meetings and sending emails.

In reference to engaging key stakeholders in programming, SWT expressed their success with changing perceptions of students; many reported how strategies such as taste tests, physical activity breaks, and monitoring screen time at home fostered behavior change in students and enhanced awareness of the importance of health behavior. The integration of students as leaders of wellness programming was described by many as a way to bolster their efforts to deliver SWITCH school-wide, as students also served as implementation champions, expanding SWT reach across the school setting. This aligns with findings from the checkpoint survey, showing that students were one of the most engaged stakeholders (see Fig. 4). Reports of parent involvement were somewhat mixed. Although some schools were successful in generating awareness, some SWT reported an overall lack of parental involvement with SWITCH programming. In relation to external change agents, SWT were able to engage local organizations, such as grocery stores and representatives from non-profit/government organizations (i.e., AmeriCorps, Food Corps) to facilitate with programming and even visit the school to help implement lessons and assist in school-wide events. Further, SWT alluded to specific strategies and practices adopted by their local Extension officer that greatly facilitated programming across the school.

With regard to executing the intervention according to plan (i.e., program best practices), several specific approaches were mentioned throughout the implementation phase as particularly successful. Many SWT found that the lessons in modules (i.e., classroom and physical education) were too long to implement (~ 20 min) or they didn’t have enough time to plan, therefore modified lessons to fit their schedule or split them up over two content periods. Other practices included teaching lessons in other parts of the school curriculum, such as science or social studies, which worked especially when school days were missed due to inclement weather. Interactive posters in the classroom, lunchroom, and physical education setting were commonly used and served to create a culture of health by driving student interest through posting trivia questions and challenges.

Finally, for the reflecting and evaluating construct, a key emphasis toward the end of SWITCH implementation was on sustainability, and how programs/practices could be sustained once the formal 12-week implementation phase was over. Many SWT felt confident that the lessons, resources, and programming would be easy to sustain and discussed how they had begun to link programming to their school wellness policy. Many tied this within their tri-annual assessment phase as per policy mandates, and found it was an important step, which allowed them to revisit many practices and policies in existence. On the other hand, some SWT felt that their policy was not a priority, and was merely a document to satisfy external requirements by administration. This was often due to administration who were either not supportive or aware of the need to revise wellness policies because of SWITCH implementation.

## Discussion

The study provides novel insights about school wellness implementation and the factors within and outside the school environment that affected school capacity and SWITCH program implementation. The adoption of CFIR and the use of both quantitative and qualitative methods approaches were critical features for studying the complex relationships influencing school wellness programming. Overall, SWT were able to adapt the intervention to meet the needs of their school setting and stakeholders, demonstrating the adaptability of SWT to mitigate structural barriers in the school environment. Findings from surveys and checkpoint calls demonstrated moderate- to-strong implementation of the best practices and an overall increase in setting-specific implementation over the 12-week period. Unlike prior studies that showed organizational readiness/capacity as a key predictor of implementation [45, 46], our findings indicated that changes in capacity followed a sequential pattern in relation to implementation, whereby the moderate and high implementing groups demonstrated greater capacity change in multiple school settings. These results have implications for further study of the relation between implementation and capacity, and the possibility of a bidirectional relation between these constructs.

Schools reported moderate-to-high engagement from students, classroom teachers, and physical education teachers, but lower engagement was reported from administration, food service staff, and parents. These findings may be, in part, due to the SWT representation which comprised mostly classroom and physical education teachers. A key finding was that school, classroom, physical education, and lunchroom capacity increased over the course of the implementation phase. These findings are reflected by those of Millar et al. [47], who evaluated the impact of implementing the “It’s Your Move” intervention in five Australian schools over a three-year period. The authors reported that capacity significantly increased in intervention schools over time, and that improvements in capacity were observed on five of the six dimensions of capacity assessed in the Community Readiness to Change tool [47].

Consistent with SWITCH, the It’s Your Move intervention [47] focused on engaging internal and external stakeholders in supporting school and school staff efforts to implement wellness initiatives. In SWITCH, community and parent engagement were enhanced by child health specialists affiliated with local 4-H County Extension offices. Specifically, Extension staff checked in with schools to provide support at several points during implementation. Through these touchpoints, Extension staff helped schools work through implementation barriers by providing advice, information on strategies that had been effective in other schools, and in some cases, they directly assisted with special events or programming.

Findings from interviews document the additive benefit of extension support; many schools felt that 4-H Extension officer was instrumental to implementation and would not have been able to implement SWITCH as well without this support. Interestingly, this type of support is often lacking in schools trying to implement wellness programming but was regarded as fitting within the scope of the 4-H Extension staff scope of responsibilities for their position [29]. The provision of funding for (or direct staffing of) a wellness coordinator is potentially effective but not sustainable [48]. Establishing effective linkages between schools and community partners that can assist with wellness program implementation in a sustainable manner is crucial for establishing long-term, effective wellness programming.

The SWRA provided a means to understand ‘Characteristics of Individuals’ and ‘Inner Setting’ factors which may contextualize the implementation practices adopted by schools. Previous studies have investigated the association between school capacity for wellness and program implementation and outcomes [45, 47, 49]. Findings from these studies document the potential importance of capacity as an influential factor associated with the implementation of school wellness initiatives. A retrospective study evaluating implementation of the CATCH intervention in 36 Illinois elementary schools identified that organizational readiness (i.e., capacity) was a key factor facilitating or hindering school staff implementation as well as classroom teacher implementation in the classroom setting [49]. The authors reported that classroom teachers were required to do more work to implement CATCH compared to physical education and cafeteria supervisors/managers and there was no direct or external incentive for their efforts [49].

In relation to ‘compatibility’ and ‘relative priority’ from the inner setting, interview data provided valuable contextual information on school capacity to implement programming. One reason why some schools reported lower capacity at baseline may relate to the finding that SWT felt they were overwhelming staff with asking them to implement SWITCH lessons or use posters in each of the school settings, as reported tension over changing the “status quo”. Some schools also reported difficulty gaining staff buy in; such tension has been reported in prior research [4, 50] and highlights complexity when a new innovation is introduced to school settings. To combat this issue, some SWT reported using staff wellness challenges to gain buy-in and enhance the overall school culture, and making SWITCH modules/resources easier to use through creating binders/photocopying lessons for teachers to take (Planning- Striking a balance). Strategies such as these demonstrate the adaptability of SWITCH materials and messaging to align with the needs and context of different school environments, ultimately enhancing implementation and systems change for wellness promotion.

In the present study, classroom teachers had the lowest level of implementation in the early phase of SWITCH compared to the lunchroom and physical education, but the highest level of implementation in the later phase of SWITCH. This may be due to the teachers having more time to explore the SWITCH module and plan for integrating the content within their curriculum and teaching schedule. In addition, the check-ins with Extension staff at the mid-point of the intervention may have been useful for helping teachers critically consider and plan for better integration of the SWITCH module during the second phase of the intervention. Interview data also highlight the positive impact of concrete ‘planning’ (implementation process) opportunities, such as the training conference and pre-implementation webinars, on implementation. This gave SWT a chance to learn vicariously from other schools’ successes (outer setting – ‘peer pressure’) and meet as a team to plan wellness initiatives for the upcoming implementation phase.

However, other factors within the inner setting (available resources), such as lack of time and facilities/personnel, hindered implementation. These barriers are highly cited issues in many school-based health behavior interventions [51–54], and in this study manifested as a perceived lack of time to implement SWITCH quality elements and best practices. SWITCH emphasizes the importance of regular SWT meetings and evaluation procedures including meetings as a component of implementation measurement; checkpoint surveys and interviews did suggest that SWT met regularly as a team to plan and coordinate implementation strategies. Through interviews, schools discussed their adaptation of this best practice by meeting briefly before/after school, within mutual planning time, and/or planning implementation through email. As such, although time is often cited as a barrier, the in-depth data analysis process facilitated understanding of how schools overcome these issues to maintain a high degree of implementation fidelity. Thus, it may be argued that time was indeed not a barrier for most SWT, more that they had competing priorities for their time (i.e., core academic subjects). When asked about how they managed to meet as a team and implement best practices, many SWT were able to negotiate their schedules to implement the program to a sufficient quality. Such findings provide practical strategies for developing school-based implementation frameworks based on empirical evidence.

With regard to ‘executing’ (implementation process), SWT reported ways in which the SWITCH materials were adapted to meet their school needs, such as teaching classroom modules in science classes, integrating the physical education module into warm-up lessons, and other practical strategies which would not have been gleaned through checkpoint surveys alone. Other researchers have found that fidelity of implementation of HIV-prevention intervention curriculum was strongly associated with student HIV knowledge and self-efficacy outcomes [55]. In their study, Wang et al. [55] categorized teachers into low, moderate, and high implementers based on their usage (number of lessons) and fidelity (carrying out lessons as planned) of curriculum indicating that fidelity to lesson plans was of great importance. In the present study, due to the comprehensive nature of SWITCH and that its quality elements/best practices target many aspects of the school environment, “fidelity” is a complex and multifaceted concept. The fact that SWT were able to adapt content to meet the needs of their individual school contexts, we believe, is a positive finding and aligns with the CFIR Intervention Characteristics construct of “adaptability” [19, 21]. Furthermore, when working with schools, it is critical to understand the pedagogical relevance of intervention materials in order to meet the needs of a wide range of school stakeholders.

Finally, the ways in which SWT reflected on implementation and the degree to which SWITCH programming could be sustained provided valuable information for future improvement. Most schools were extremely positive in their perceptions of SWITCH as a sustainable program, yet when discussing implications for their school or district wellness policy, several SWT had little to no involvement in this process. Furthermore, some reported that, when asking about their policy, were not given clear information on this document or its whereabouts, highlighting a disconnect between policy and practice [56]. Although training was provided at the in-person conference about the Final Rule mandate, it is likely that more training and support is needed to help SWT as a means to enhance the connection between practice and policy.

The findings add rich understanding to the steps needed to sustain school wellness programming in school settings [6, 23], but it is important to acknowledge limitations. First, the SWITCH schools self-selected to enroll in the program, and were not representative of all schools in the state or country, limiting generalizability of these findings. Second, the checkpoint surveys and interviews provided self-reported (i.e., school-reported) implementation data and may be subject to bias on behalf of schools. It is important to consider the methods of data collection within D&I science, as researchers must strike a balance between collecting high-quality implementation data without over-burdening school sites [57, 58], thus self-report data may present the most feasible method for obtaining rich information from stakeholders. Finally, the checkpoint calls and interviews were conducted at times convenient for SWT members, and sometimes not all group members could not be present, limiting our understanding of all aspects of implementation. However, it must be noted that this is a logistical challenge when working with schools and it is imperative to adopt flexible approaches to data collection in such dynamic settings.

## Conclusions

The purpose of this study was to examine the factors within and outside the school environment that influence capacity for program implementation. Findings underscore the need to study implementation using multiple methods, and the organizational capacity that might predict the degree to which schools implement the program. Multiple constraints were found to arise from the inner setting, such as lack of time/resources, staff buy-in, and administrator support. However, the provision of 4-H Extension support (outer setting) and the adaptability of the SWITCH curriculum/resources (intervention characteristics, implementation process) to be tailored to meet school context and capacity. This additional support may mitigate some of the barriers and constraints faced by SWT, enhancing the potential for sustainability of SWITCH. Finally, results show the importance of the school wellness conference as a training and socialization process, acting as a catalyst for planning and readiness for implementation. Further improvement to the SWITCH program is needed to support schools in taking their practice to policy, as a means to enhance school wellness environments and promote student health behavior.

## Supplementary Information

**Additional file 1: Appendix A.** School Wellness Readiness Assessment Tool (SWRA).

**Additional file 2: Appendix B.** Interview Guides.

**Additional file 3: Appendix C.** Consolidated Framework for Implementation Research (CFIR) Codebook.

**Additional file 4.** StaRI- Implementation Checklist.

**Additional file 5.** COREQ Checklist.

## Data Availability

We have made the qualitative codebook and the interview guides for further information.
