# Supplementary material for: Evaluating the implementation of the SWITCH® school wellness intervention and capacity-building process through multiple methods
Source: Int J Behav Nutr Phys Act. 2020 Dec 11;17:162. doi: 10.1186/s12966-020-01070-y (PMC7733251; doi:10.1186/s12966-020-01070-y)
Supplement: Supplementary file 1 — Additional file 1: Appendix A. School Wellness Readiness Assessment Tool (SWRA). [file 12966_2020_1070_MOESM1_ESM.pdf]

School Wellness Readiness Assessment Tool

The School Wellness Readiness Assessment (SWRA) tool is used to measure your school's readiness for change. Completion of the tool should be facilitated by the school's wellness team. The wellness team should engage staff/colleagues from each of the key settings to complete each section of the tool.

It is important to provide an honest assessment for each of the items to best understand the status of readiness for change and capacity for comprehensive wellness programming in the school. It is also important to ensure that the opinions of all staff members are captured and reflected in the responses and to not only include input from those eager and willing to participate.

It is OK if some scores are lower than others. Understanding the readiness for change within each area of the school can help with understanding why change may occur within the school at different rates and help to tailor programming or training to meet the needs and capacity of the school and staff.

The organizational items ask about the school's overall vision and leadership. The individual items ask about the staffs' individual perceptions of the school's readiness for change.

Individual Structural					
Please respond to the following statements about the extent to which staff in each area possess the knowledge, skills, and capacity to carry out school wellness initiatives.					
**Classroom Teachers**	Strongly Disagree	Disagree	Neither Agree or Disagree	Agree	Strongly Agree
1. Are well-informed on strategies to support wellness within the classroom.					
2. Have the skills necessary to deliver health education to students and lead classroom activities.					
3. Are given the opportunity to implement health education topics and classroom activity breaks in their lesson plans.					
4. Are provided with resources to implement health education curriculum and classroom activity breaks.					
5. Are provided with opportunities for professional development on integrating health education curriculum and classroom activity breaks in their classes.					
**Physical Education Teachers**	Strongly Disagree	Disagree	Neither Agree or Disagree	Agree	Strongly Agree
6. Are well-informed on Health Literacy and strategies for promoting activity throughout and outside of school.					
7. Have the skills necessary to integrate school wellness programming during and surrounding the school day.					
8. Are given the opportunity to implement school wellness programs/initiatives.					
9. Are provided with resources to implement quality Physical Education/school wellness programming.					
10. Are provided with opportunities for professional development on Physical Education/school wellness programming.					
**Food Service Personnel**	Strongly Disagree	Disagree	Neither Agree or Disagree	Agree	Strongly Agree
11. Are well-informed on best practices for promoting healthy eating in the lunchroom and throughout the school.					
12. Have the skills necessary to carry out best practices for promoting healthy eating in the lunchroom and throughout the school.					
13. Are given the opportunity to implement best practices for promoting healthy eating in the lunchroom and throughout the school.					
14. Are provided with resources to implement strategies to promote healthy eating in the lunchroom and throughout the school.					
15. Are provided with opportunities for professional development on strategies for promoting healthy eating in the lunchroom and throughout the school.					

Individual Psychological					
Please respond to the following statements about the extent to which staff in each area of the school hold beliefs towards the need for and importance of school wellness initiatives.					
**Classroom Teachers**	Strongly Disagree	Disagree	Neither Agree or Disagree	Agree	Strongly Agree
16. Believe that classroom wellness initiatives can directly influence student health.					
17. Believe that integrating health education and wellness activities (e.g., activity breaks) are worthwhile uses of classroom time.					
18. Believe that school leadership is committed to supporting school wellness initiatives in the classroom.					
19. Believe that wellness education and opportunities belong in the classroom setting.					
20. Believe that wellness education and opportunities can be implemented in classrooms successfully.					
**Physical Education Teachers**	Strongly Disagree	Disagree	Neither Agree or Disagree	Agree	Strongly Agree
21. Believe that incorporating health and behavioral education in Physical Education can directly influence student health.					
22. Believe that including health and behavioral education in lesson plans is a worthwhile use of Physical Education time.					
23. Believe that integrating school wellness efforts during and surrounding the school day is a worthwhile pursuit.					
24. Believe that school leadership is committed to supporting Physical Education.					
25. Believe that school leadership is committed to supporting school wellness efforts during and surrounding the school day.					
**Food Service Personnel**	Strongly Disagree	Disagree	Neither Agree or Disagree	Agree	Strongly Agree
26. Believe that incorporating lunchroom and school food environment initiatives can directly influence student health.					
27. Believe that integrating lunchroom and school food environment strategies are a worthwhile use of time.					
28. Believe that school leadership is committed to supporting school nutrition initiatives.					
29. Believe that school food environment initiatives are roles that belong to food service and lunchroom staff.					
30. Believe that lunchroom and school food environment strategies can be implemented in the school successfully.					

Organizational Structural					
Please respond to the following statements about the school's climate and environment towards school wellness initiatives.	Strongly Disagree	Disagree	Neither Agree or Disagree	Agree	Strongly Agree
31. The school provides sufficient resources (staff, equipment) for fostering a healthy school environment.					
32. School leadership provide a clear vision about the school's plan for pursuing wellness-related goals.					
33. School leadership clearly define staffs' roles/responsibilities as it relates implementing the school's wellness plan.					
34. School leadership provides a consistent message of support for pursuing/implementing wellness-related initiatives.					
35. The school environment/culture always prioritizes healthy choices.					

Organizational Psychological					
Please respond to the following statements about the school and school staffs' collective school wellness perceptions.	Strongly Disagree	Disagree	Neither Agree or Disagree	Agree	Strongly Agree
36. Staff members at all levels within the school work together effectively on school wellness initiatives.					
37. Staff members at all levels share a belief that they can implement school wellness programs effectively.					
38. Staff members at all levels have a collective commitment to support school wellness initiatives.					
39. Staff members at all levels share a collective resolve to ensure that school wellness initiatives are successful in the school.					
40. Staff members at all levels share a collective belief that wellness initiatives are worthwhile pursuits.					

Submitting Results and Conclusion

Please email your completed SWRA to Joey Lee, at jlee29@uccs.edu or via standard mail to:

University of Colorado at Colorado Springs
University Hall Room 202A
1420 Austin Bluffs Pkwy
Colorado Springs, CO 80918
Attn: Joey Lee

Your school's results will be reported back to you for informing the status of your school readiness for change. The results can be used strategically to tailor potential programming the school is considering or developing or to identify potentially effective professional development opportunities for staff and administrators.

Thank you for taking the time to complete the School Wellness Readiness Assessment Tool!
