# Supplementary material for: Evaluating the implementation of the SWITCH® school wellness intervention and capacity-building process through multiple methods
Source: Int J Behav Nutr Phys Act. 2020 Dec 11;17:162. doi: 10.1186/s12966-020-01070-y (PMC7733251; doi:10.1186/s12966-020-01070-y)
Supplement: Supplementary file 2 — Additional file 2: Appendix B. Interview Guides. [file 12966_2020_1070_MOESM2_ESM.docx]

**Interview Guides for Qualitative Data Collection**

This attachment contains:

- Discussion/MI interview script for checkpoint calls with core teams (mid-and end-point)
- Interview guide for core teams post-SWITCH
- Interview guide for extension staff post-SWITCH

**Checkpoint Call Interview Guide (Week 6 call)**

1. Tell me what point you are at with SWITCH in your school.
   1. What kind of progress have you made with implementing the best practices/quality elements?
   2. Which have been easier to implement?
   3. Which have been more difficult to implement?
2. What successes have you experienced with the program? Tell me about these and how these successes were achieved.
3. What has been difficult or challenging for you so far?
   1. Were these challenges environmental (i.e. schedule, school building) or interpersonal (difficulty getting buy-in from others, lack of interest from teachers, etc.)?
4. Let’s talk about the goals for SWITCH that you set back at the beginning (mention goals here). What progress have you made toward these goals?
   1. Were these goals realistic or too challenging?
   2. What successes and challenges have you experienced in putting these goals into action?
   3. How competent did you feel in implementing these goals?
   4. What kind of support did you have with implementing these goals?
5. Let’s talk about the school wellness environment. What changes, if any, have you seen in your school environment or culture?
   1. What are the main areas you identified as priorities from the SWEP report (include priority areas here)?
   2. Can you tell me what steps have been taken to improve these areas?
   3. How do you think the school environment will facilitate/impede these changes?

**Final Checkpoint Call Interview Guide (Led by Extension)**

**Checking in with Schools**

1. Tell me about SWITCH implementation at your school these past 6 weeks
   1. What have been your team’s major achievements?
   2. What were some barriers that you faced in the process?
      1. How did your team work to overcome these barriers?
2. Let’s talk about the goals you set at the beginning of SWITCH. How much progress do you feel you have made toward these goals?
   1. What factors facilitated this progression? Why?
   2. What factors hindered this progression? Why?
   3. Looking back on these goals, to what degree do you think they were realistic?
   4. If you were to set goals again, what would you do differently?

**Motivational Interview**

1. Let’s talk about your SWEP. What major changes have you noticed in your school wellness environment?
   1. (if pre-post report available) look at the areas of change. Tell me about these areas of biggest improvement. Why do you think these changes occurred?
      1. What led to these changes?
      2. What challenges did you face in making these changes and how did you overcome them?
2. Think of the changes you have made in your school setting. To what degree do you think these changes are sustainable?
3. What do you think the next steps are for improving your school wellness environment?
   1. What goals can you set for the next few months?
   2. How are you going to achieve these goals?
4. What about your school wellness policy? How do you think the work you have done in SWITCH can relate to your wellness policy?
5. Let’s talk about these goals for the future. Do you think they are feasible? Why/why not? If not, let’s talk about what changes could be feasible for your school wellness environment/policy
6. Are there any other ideas you have for your wellness environment? Let’s talk about those briefly
7. What other questions/comments do you want to add?

**School Core Team Interview Guide (CSSM)**

1. Tell me about overall SWITCH implementation at your school.
   1. What were your initial perceptions of the program?
   2. Have these perceptions change at all over the course of SWITCH implementation?
   3. If so, what factors impacted your views on the program?
2. How did you perceive the utility of the training received prior to program implementation?
   1. Webinars (pre-conference and post-conference)
   2. SWITCH school wellness conference (November 2018)
   3. First checkpoint call (January 2019)
3. What would you say was the most beneficial aspect of training for your school?
   1. Why was this? How did it help your core team succeed in SWITCH?
4. What would you say were some challenges faced by your school?
   1. Why was this? How did you address these?
5. How would you describe the level of support you received from your county youth extension officer(s)?
   1. To what degree did they facilitate SWITCH implementation?
   2. What was the nature of your communication with your extension officer? How often did you meet and what were some of the main aspects of SWITCH you discussed?
   3. How might this support system be improved for future iterations of SWITCH?
   4. Imagine that extension staff were not involved at all this year. How do you think SWITCH would look in your school?
6. How would you describe the level of support you received from the SWITCH team at Iowa State?
   1. To what degree did they facilitate SWITCH implementation?
   2. How did you feel about the level of communication you received from the SWITCH team? To what degree were weekly emails beneficial to your core team’s success?
   3. What aspects of communication/support were most beneficial to your school?
   4. How could the SWITCH team’s involvement be improved?
7. The goal of the ISU SWITCH team is to disseminate the program so that all schools across the state have the opportunity to engage in this initiative. What would you tell teachers at other schools if they asked you about implementing SWITCH at their school?
8. If your school had the opportunity to do SWITCH again this year, is this something you would be interested in?
9. On a scale from 1-5, how likely are you to recommend SWITCH to another school?
10. On a scale from 1-5, how beneficial was SWITCH for your school wellness environment?
11. On a scale from 1-5, how engaged and supportive was your county extension staff member?
