# Supplementary material for: Evaluating the implementation of the SWITCH® school wellness intervention and capacity-building process through multiple methods
Source: Int J Behav Nutr Phys Act. 2020 Dec 11;17:162. doi: 10.1186/s12966-020-01070-y (PMC7733251; doi:10.1186/s12966-020-01070-y)
Supplement: Supplementary file 3 — Additional file 3: Appendix C. Consolidated Framework for Implementation Research (CFIR) Codebook. [file 12966_2020_1070_MOESM3_ESM.docx]

SWITCH Qualitative CFIR Codebook

Nodes

| Name | Description | Files | References |
| --- | --- | --- | --- |
| CFIR Constructs |  | 0 | 0 |
| I. INNOVATION CHARACTERISTICS | Characteristics of the intervention being implemented into a particular organization. Ideally, we'd have a definition of the "essential core" components of the intervention versus the "flexible periphery." | 0 | 0 |
| A. Innovation Source | Perception of key stakeholders about whether the intervention is externally or internally developed. | 1 | 1 |
| B. Evidence Strength & Quality | Stakeholders’ perceptions of the quality and validity of evidence supporting the belief that the intervention will have desired outcomes. | 0 | 0 |
| C. Relative Advantage | Stakeholders’ perception of the advantage of implementing the intervention versus an alternative solution. | 0 | 0 |
| D. Adaptability | The degree to which an intervention can be adapted, tailored, refined, or reinvented to meet local needs. | 0 | 0 |
| Adaptive Implementation Strategies | Core Teams discussed their use of lunchroom, PE, and classroom modules and the ways in which they successfully implemented them despite pressures of time, lack of training, etc. | 26 | 34 |
| E. Trialability | The ability to test the intervention on a small scale in the organization [8], and to be able to reverse course (undo implementation) if warranted. | 0 | 0 |
| F. Complexity | Perceived difficulty of implementation, reflected by duration, scope, radicalness, disruptiveness, centrality, and intricacy and number of steps required to implement | 28 | 35 |
| G. Design Quality & Packaging | Perceived excellence in how the intervention is bundled, presented, and assembled | 2 | 2 |
| H. Cost | Costs of the intervention and costs associated with implementing that intervention including investment, supply, and opportunity costs. | 0 | 0 |
| II. OUTER SETTING | Generally, the outer setting includes the economic, political, and social context within which an organization resides. Changes in the outer setting can influence implementation, often mediated through changes in the inner setting. | 0 | 0 |
| A. Needs & Resources of Those Served by the Organization | The extent to which patient needs, as well as barriers and facilitators to meet those needs are accurately known and prioritized by the organization. | 0 | 0 |
| B. Cosmopolitanism | The degree to which an organization is networked with other external organizations. | 0 | 0 |
| Collaborating with Community and Organizations | Schools reported their collaboration with community stakeholders and organizations. They reported their efforts to reach out to grocery stores, non-profit organizations, and other entities, and the relative successes they had while engaging with these individuals. | 21 | 37 |
| Extension Support | A primary theme of extension support to school-level SWITCH implementation | 0 | 0 |
| Accountability for Goal Setting | Staff helped schools stay accountable for their progress and goals by keeping focus specific to initial goals. Many schools commented that this kept them on track. | 17 | 25 |
| Lack of Extension Support | Schools reported a lack of extension support for a variety of reasons (i.e., low contact, not sure who to ask for help etc.) This either did not affect implementation or negatively impacted it. | 6 | 7 |
| Part of the team | Extension staff often provided supporting statements to the team during checkpoint calls and offering to assist with programming. During end-point interviews schools expressed how critical extension staff were to the program. | 18 | 25 |
| Provision of Ideas and resources | During checkpoint calls extension staff were often instigators of ideas and shared resources to help teams work through problems. | 32 | 50 |
| C. Peer Pressure | Mimetic or competitive pressure to implement an intervention; typically because most or other key peer or competing organizations have already implemented or in a bid for a competitive edge. | 6 | 6 |
| Administrator Support | School core teams reflected on support/lack of support from school administration. This manifested differently between schools and some reported more implicit types of support, such as scheduling allowances; other more explicit kinds of support were also documented such as principals taking part in programs or being on the committee. | 6 | 6 |
| D. External Policy & Incentives | A broad construct that includes external strategies to spread interventions including policy and regulations (governmental or other central entity), external mandates, recommendations and guidelines, pay-for-performance, collaboratives, and public or benchmark reporting. | 7 | 7 |
| Awareness of local and national policy to push change | School core teams felt a sense of obligation to implement SWITCH given external pressures such as policy at the national or state level. They may also have observed changes in overall health of students and internalized this as a means to improve. | 3 | 3 |
| III. INNER SETTING | Includes features of structural, political, and cultural contexts through which the implementation process will proceed. May be composed of tightly or loosely coupled entities (e.g., a loosely affiliated medical center and outlying contracted clinics or tightly integrated service lines within a health system); tangible and intangible manifestation of structural characteristics, networks and communications, culture, climate, and readiness all interrelate and influence implementation. | 0 | 0 |
| A. Structural Characteristics | The social architecture, age, maturity, and size of an organization. | 1 | 1 |
| B. Networks & Communications | The nature and quality of webs of social networks and the nature and quality of formal and informal communications within an organization. | 0 | 0 |
| Between core team and school collaborations | Schools reported working directly with other members of the school community, such as PE teachers, lunchroom, classroom, and nurses to implement programming and found it to facilitate overall implementation | 22 | 27 |
| Lack of staff buy-in | Some core teams struggled to engage staff members in these initiatives and lamented their frustration as this negatively impacted implementation | 31 | 55 |
| Shared versus sole leadership | Through interviews and checkpoint calls, the topics of “collaboration” and “leadership” arose. While many core teams were designed as a group it was often one person leading implementation | 20 | 34 |
| Success with staff buy-in | It was hard to get staff on board, especially when schools were doing SWITCH for the first time. Many expressed that their policies/programs would not be favorably view by their colleagues. | 48 | 99 |
| C. Culture | Norms, values, and basic assumptions of a given organization. | 0 | 0 |
| Lack of prior experience posed challenges | New core teams stressed that being new to SWITCH was difficult and that they found it hard to implement best practices since there was so much to focus on. | 15 | 20 |
| Overwhelming Staff | Core teams spoke of not wanting to “overwhelm staff” or step on toes, and did not feel comfortable presenting SWITCH to their school. Others reported that some staff were not on board or provided barriers to buying in to the model. | 30 | 58 |
| SWITCH Experience facilitated implementation | Schools lamented their increased capacity now they have experienced SWITCH a second time around, compared to newer schools. Many of them stated their first year was not very successful and they needed a whole year to “get the hang of it” | 27 | 46 |
| SWITCH Experience Negative Case | Schools that were experienced but still reported challenges with implementation. | 3 | 4 |
| D. Implementation Climate | The absorptive capacity for change, shared receptivity of involved individuals to an intervention and the extent to which use of that intervention will be rewarded, supported, and expected within their organization. | 0 | 0 |
| 1. Tension for Change | The degree to which stakeholders perceive the current situation as intolerable or needing change. | 2 | 2 |
| Awareness of local and national policy to push change | School core teams felt a sense of obligation to implement SWITCH given external pressures such as policy at the national or state level. They may also have observed changes in overall health of students and internalized this as a means to improve. | 3 | 3 |
| Wellness Policy | Schools discuss ways that their SWITCH programming links to their wellness policy | 14 | 21 |
| 2. Compatibility | The degree of tangible fit between meaning and values attached to the intervention by involved individuals, how those align with individuals’ own norms, values, and perceived risks and needs, and how the intervention fits with existing workflows and systems. Code instances of duplication with existing systems (e.g., between MMT and CPRS, other patient outreach initiatives that target the same patients as AIM) | 0 | 0 |
| Tension for changing norms | Tension occurs when some norms such as withholding recess for academics/punishment or letting students bring in cake for birthdays do not align with the SWITCH best practices | 17 | 25 |
| 3. Relative Priority | Individuals’ shared perception of the importance of the implementation within the organization. | 4 | 5 |
| Active promotion and priority of SWITCH | Reports of active promotion and priority for SWITCH from principals and teachers. Examples include (but not limited to) change of scheduling, increased opportunity for activity, and school-wide initiatives. | 8 | 9 |
| 4. Organizational Incentives & Rewards |  | 0 | 0 |
| Increased social capital | Core team members report their hopes/experiences of increased social capital for implementing SWITCH. Sources could come from other teachers but mainly administration | 1 | 1 |
| 5. Goals and Feedback |  | 0 | 0 |
| Reflection of Goals | Core teams report reflection of goals and progress toward such benchmarks as a means to facilitate implementation | 2 | 2 |
| 6. Learning Climate |  | 0 | 0 |
| Enhancing the culture of wellness among staff and students | School-wide initiatives such as step challenges were introduced and teams commented that they noticed heightened engagement during checkpoint calls and end-point interviews. | 39 | 94 |
| Overwhelming Staff | Core teams spoke of not wanting to “overwhelm staff” or step on toes, and did not feel comfortable presenting SWITCH to their school. Others reported that some staff were not on board or provided barriers to buying in to the model. | 25 | 52 |
| E. Readiness for Implementation | Tangible and immediate indicators of organizational commitment to its decision to implement an intervention. | 0 | 0 |
| 1. Leadership Engagement |  | 0 | 0 |
| Administrator Support | School core teams reflected on support/lack of support from school administration. This manifested differently between schools and some reported more implicit types of support, such as scheduling allowances; other more explicit kinds of support were also documented such as principals taking part in programs or being on the committee. | 26 | 45 |
| Administrator Support (2) | School core teams reflected on support/lack of support from school administration. This manifested differently between schools and some reported more implicit types of support, such as scheduling allowances; other more explicit kinds of support were also documented such as principals taking part in programs or being on the committee. | 25 | 44 |
| Lack of Administrator support | Schools may report less/lack of support from their administrator, hindering SWITCH implementation | 8 | 10 |
| Students playing a role | Students were involved in leading some aspects of SWITCH through 4H/youth ambassadors clubs, playing a strong role in implementation | 37 | 76 |
| Students playing a role (2) | Students were involved in leading some aspects of SWITCH through 4H/youth ambassadors clubs, playing a strong role in implementation | 17 | 25 |
| 2. Available Resources |  | 0 | 0 |
| Lack of available resources | Time, money, space- all factors that Core Teams cite as negatively influencing implementation | 23 | 34 |
| 3. Access to Knowledge and Information |  | 1 | 1 |
| Online platform can be a help and a hindrance | Although SWITCH team set up the website as a means to collect data easily and efficiently and engage schools, some expressed that they were confused by the platform and its features, leading to less utilization. | 31 | 38 |
| Overwhelming Staff | Core teams spoke of not wanting to “overwhelm staff” or step on toes, and didn’t feel comfortable presenting SWITCH to their school. Others reported that some staff were not on board or provided barriers to buying in to the model. | 27 | 51 |
| Provision of Ideas and resources | During checkpoint calls extension staff were often instigators of ideas and shared resources to help teams work through problems. | 31 | 51 |
| IV. INDIVIDUAL CHARACTERISTICS | Individuals involved with the intervention and/or implementation process. Code specific roles related to champion, opinion leaders, formally appointed implementation leaders, and external change agents under the appropriate code under PROCESS. Mostly, these codes involve people who are the target of the intervention (primarily users). | 0 | 0 |
| A. Knowledge & Beliefs | Individuals’ attitudes toward and value placed on the intervention as well as familiarity with facts, truths, and principles related to the intervention. | 1 | 1 |
| B. Self-efficacy | Individual belief in their own capabilities to execute courses of action to achieve implementation goals. | 0 | 0 |
| C. Individual Stage of Change | Characterization of the phase an individual is in, as he or she progresses toward skilled, enthusiastic, and sustained use of the intervention. | 0 | 0 |
| D. Individual Identification with Organization | A broad construct related to how individuals perceive the organization and their relationship and degree of commitment with that organization. INCLUDE Organizational citizenship: how organizational identity is taken on and whether individuals are willing to put in extra effort, talk well of the organization, and take risks in their organization. Organizational justice: perception of distributive and procedural fairness in the organization. Emotional exhaustion: burnout. Etc. | 0 | 0 |
| Between core team and school communication | Core teams reported the different ways they successfully communicated with other staff, leading to greater reach across the school and buy-in from teachers. | 19 | 31 |
| Shared versus sole leadership | Through interviews and checkpoint calls the topics of “collaboration” and “leadership” arose. While many core teams were designed as a group it was often one person leading implementation | 16 | 30 |
| E. Other Personal Attributes | A broad construct to include other personal traits such as tolerance of ambiguity, intellectual ability, motivation, values, competence, capacity, and learning style. | 0 | 0 |
| High Core Team Motivation | Certain schools reported high levels of motivation to implement SWITCH and specifically addressed their passion for making positive change as a strengthening factor for implementation | 22 | 37 |
| Low Core Team Motivation, Preparation | Some core teams reported lower motivation/excitement for implementing SWITCH and this was linked to their lack of ability to get started and engage others in their school | 0 | 0 |
| High Core Team Motivation (2) | Certain schools reported high levels of motivation to implement SWITCH and specifically addressed their passion for making positive change as a strengthening factor for implementation | 16 | 21 |
| Low Core Team Motivation, Preparation | Some core teams reported lower motivation/excitement for implementing SWITCH and this was linked to their lack of ability to get started and engage others in their school | 9 | 13 |
| V. PROCESS | Activities aimed to achieve individual and organizational level use of the intervention as designed. Process may be an interrelated series of sub-processes that do not necessarily occur sequentially. These sub-processes may be formally planned or spontaneous; conscious or subconscious; linear or nonlinear. | 0 | 0 |
| A. Planning | The degree to which a scheme or method of behavior and tasks for implementing an intervention are developed in advance and the quality of those schemes or methods. | 0 | 0 |
| Core Team Weekly Meetings | Core teams' reported meeting on a weekly basis | 16 | 19 |
| Importance of wellness conference | Schools demonstrate the importance of attending the wellness conference as a means to facilitate their planning efforts. | 17 | 22 |
| Overwhelming Staff | Core teams spoke of not wanting to “overwhelm staff” or step on toes, and didn’t feel comfortable presenting SWITCH to their school. Others reported that some staff were not on board or provided barriers to buying in to the model. | 24 | 50 |
| B. Engaging | Attracting and involving appropriate individuals in the implementation and use of the intervention through a combined strategy of social marketing, education, role modeling, training, and other similar activities. | 0 | 0 |
| 1. Opinion Leaders | Individuals in an organization who have formal or informal influence on the attitudes and beliefs of their colleagues with respect to implementing the intervention | 0 | 0 |
| Administrator Support | School core teams reflected on support/lack of support from school administration. This manifested differently between schools and some reported more implicit types of support, such as scheduling allowances; other more explicit kinds of support were also documented such as principals taking part in programs or being on the committee. | 24 | 43 |
| Changing Students' Perceptions | Core teams spoke of the ways in which they experienced “aha moments” with students through using modules, learning activities, or school-wide events, etc. | 32 | 58 |
| Success Engaging Parents in Programming | Email communication was cited as a primary way to engage parents, with some schools taking an extra step to include them in programming. | 16 | 23 |
| Difficulty Engaging Parents | School core teams reported difficulty engaging community and parents | 14 | 15 |
| Plans to engage parents |  | 23 | 26 |
| 2. Formally Appointed Implementation Leader | Individuals from within the organization who have been formally appointed with responsibility for implementing an intervention as coordinator, | 0 | 0 |
| 3. Champion | “Individuals who dedicate themselves to supporting, marketing, and ‘driving through’ an [implementation],” overcoming indifference or resistance that the intervention may provoke in an organization. | 0 | 0 |
| Shared versus sole leadership | Through interviews and checkpoint calls the topics of “collaboration” and “leadership” arose. While many core teams were designed as a group it was often one person leading implementation | 13 | 23 |
| Students playing a role | Students were involved in leading some aspects of SWITCH through 4H/youth ambassadors clubs, playing a strong role in implementation | 36 | 65 |
| Within core team communication | (linked to shared versus sole leadership) – core teams reported communication varied and most of the time teams linked up via email, with few in-person meetings occurring. | 30 | 41 |
| 4. External Change Agent |  | 0 | 0 |
| Engaging Community and Parents | Email communication was cited as a primary way to engage parents, with some schools taking an extra step to include them in programming. | 42 | 83 |
| Extension Support | A primary theme of extension support to school-level SWITCH implementation | 0 | 0 |
| Accountability for Goal Setting | Staff helped schools stay accountable for their progress and goals by keeping focus specific to initial goals. Many schools commented that this kept them on track. | 25 | 37 |
| Part of the team | Extension staff often provided supporting statements to the team during checkpoint calls and offering to assist with programming. During end-point interviews schools expressed how critical extension staff were to the program. | 24 | 33 |
| Provision of Ideas and resources | During checkpoint calls extension staff were often instigators of ideas and shared resources to help teams work through problems. | 31 | 51 |
| External Collaborations | Core teams found that partnering with companies like Hy-Vee and other local organizations were helpful in getting students and teachers on board with SWITCH. Some reported unsuccessful collaboration attempts. | 25 | 39 |
| 5. Key Stakeholders (Staff) |  | 0 | 0 |
| 6. Innovation Participants (Patients) |  | 0 | 0 |
| Changing Students' Perceptions | Core teams spoke of the ways in which they experienced “aha moments” with students through using modules, learning activities, or school-wide events, etc. | 42 | 78 |
| Difficulty engaging Teachers and other staff | Data ae coded here when they don't fit in the "complexity" theme but are related to difficulty preparing teachers and other staff. Data here don't align 100% with the lack of staff buy-in theme | 7 | 8 |
| C. Executing | Carrying out or accomplishing the implementation according to plan. | 0 | 0 |
| Quality Elements and Best Practices | Internal/logistical coding regarding use of quality elements and best practices. | 80 | 252 |
| SWITCH Posters | Core teams' usage of posters | 5 | 6 |
| SWITCH Trinkets | Core Team's usage of the trinkets to promote tracking behavior | 12 | 25 |
| Sustainability | When schools discuss wellness programming and practices as being "sustainable" long-term or when they bring up barriers to keeping things going over time. | 23 | 50 |
| Changes to School Wellness Environment | Code here any quotes that relate to programmatic changes, system changes, or any kind of practice that is being introduced that relates to Do, View, or Chew | 18 | 51 |
| Enhancing the culture of wellness among staff and students | School-wide initiatives such as step challenges were introduced and teams commented that they noticed heightened engagement during checkpoint calls and end-point interviews. | 37 | 85 |
| SWITCH Experience | Schools lamented their increased capacity now they have experienced SWITCH a second time around, compared to newer schools. Many of them stated their first year was not very successful and they needed a whole year to “get the hang of it” | 38 | 62 |
| Wellness Policy | Schools discuss ways that their SWITCH programming links to their wellness policy | 14 | 21 |

Note: Adapted based on CFIR constructs: <https://cfirguide.org/evaluation-design/qualitative-data/>
